# Digital anatomical study and clinical application of the ideal S2 alar-lliac screw trajectory

**DOI:** 10.1186/s12893-023-02167-4

**Published:** 2023-10-04

**Authors:** Yonghui Zhao, Yulong Ma, Qiyang Wang, Haotian Luo, Jie Liu, Sheng Lu

**Affiliations:** Department of Orthopedics, The First People’s Hospital of Yunnan Province, The Affiliated Hospital of Kunming University of Science and Technology, 157 Jinbi Road, Kunming, Xishan District China

**Keywords:** Spinal pelvic fixation, S2AI, Ideal screw trajectory

## Abstract

**Background:**

To investigate the ideal trajectory for the S2AI screw and to clinically validate its safety feasibility.

**Methods:**

The 3D model was reconstructed from CT data of the pelvis of 30 selected adults, and the 3D coordinate system was established with the first sacral superior endplate as the horizontal plane. A set of cutting planes was made at 3 mm intervals in the coronal plane, and the cross-sectional internal tangent circles were divided in the target area. Using the linear fitting function, the axis of 90 mm length was calculated by the least squares method for each inner tangent circle center. The diameter of the axis is gradually increased until the first contact with the cortex, and the cylindrical model is the ideal screw trajectory. The intersection of the axis and the dorsal cortex is the screw placement point, which is located by Horizon Distance (HD) and Vertical Distance (VD); the diameter of the screw trajectory (d) is the diameter of the cylindrical model; the direction of the screw trajectory is determined by Sagittal Angle (SA) and Transverse Angle (TA). The screw trajectory orientation is determined by Sagittal Angle (SA) and Transverse Angle (TA). Based on the ideal screw trajectory, the 3D printed surgical guide and freehand techniques were used to verify its safety feasibility, respectively.

**Results:**

The screw placement points [HD (4.7 ± 1.0) mm, VD (19.7 ± 1.9) mm], screw placement directions [SA (31.3°±2.3°), TA (42.4°±2.3°)], and screw dimensions for the ideal screw trajectory of the S2AI were combined for analysis. (L is 90 mm, d is 13.2 ± 1.4 mm). The S2AI screw superiority rate [96.6% (56/58)] and reasonable rate [100%] were higher in the guide group than in the freehand group [90.0% (63/70), 97.1% (68/70)], but the differences were not statistically significant (P > 0.05). Although screws invaded the cortex in both groups, there were no associated adverse events in either group.

**Conclusion:**

The S2AI screw-based ideal trajectory placement is a safe, feasible and accurate method of screw placement.

## Introduction

Spinal pelvic fixation is widely used in adult spinal deformities and degenerative scoliosis [1–3]. Due to the short and stress-concentrated screw trajectory of conventional sacral screws, they are prone to postoperative fusion failure and other related complications [4–8]. Iliac screws can improve the strength of spinal pelvic fixation [9, 10], but they are more traumatic, and the internal fixation protrudes through the skin, increasing the risk of postoperative infection [11–13]. The S2-alar-iliac screw (S2AI) screw technique compensates for these shortcomings and has become a prominent research topic in spinal pelvic fixation in recent years [14–16].

The placement trajectory of the S2AI screw enters through the posterior aspect of the sacrum, penetrates the sacroiliac joint to the iliac bone, and ends above the acetabulum. Study showed that the S2AI screw punctured the screw trajectory and could damage the sciatic nerve, obturator nerve, superior gluteal artery, internal iliac artery, and lumbosacral plexus, resulting in medically induced injuries. Therefore, the safety and accuracy of the S2AI screw trajectory are crucial [17].

Currently, S2AI screw placement methods are divided into freehand techniques and adjunctive techniques. Assistive techniques include 3D printed surgical guides, intraoperative navigation, and robotics to assist in screw placement [18–20]. Although assistive techniques have demonstrated high safety and accuracy, their limited availability restricts clinical applications. The freehand technique remains the most commonly used method. Most studies on S2AI screw trajectorys are based on two-dimensional images [21–23]. It is worth considering whether the 2D planar design of the screw trajectory can truly reflect the actual situation of the 3D structure, and there must exist an ideal screw trajectory that is most safely close to the center of the sacrum and iliac bone despite the irregularity of the passage between them. In this study, we investigated the S2AI screw trajectory based on the 3D anatomical structure using digital technology, elaborated the parameters of the ideal screw trajectory for S2AI, and further conducted clinical application to verify the safety and feasibility of the screw trajectory.

## Materials and methods

### General data

We retrospectively collected pelvic computed tomography (CT) data from 30 adult cases, all scanned by a 64-row spiral CT (GE, USA) with a layer thickness of 0.625 mm. The sample included 15 cases of each sex, with an age range of 57.9 ± 17.4 years (range: 20–65 years). Inclusion criteria were: (1) age ≥ 18 years; (2) complete pelvic CT scan data; (3) normal structure of the iliac bone, sacrum, and their attachments. Exclusion criteria included: (1) pelvic developmental deformities; (2) pelvic fractures, infections, or tumors; (3) previous history of pelvic and lumbosacral surgery.

### Research methods

#### Pelvis modeling

The CT data of the 30 patients were imported into Mimics 19.0 software in DICOM format for 3D reconstruction, generating 30 3D models of the pelvis in Stereolithograph (STL) format. The STL format data were imported into Geomagic Studio 12.0 software, where the 3D models of the pelvis underwent filling, repair, and other image optimization processes.

#### Design of the ideal screw trajectory

##### Pelvic plane cutting

**Fig. 1:**
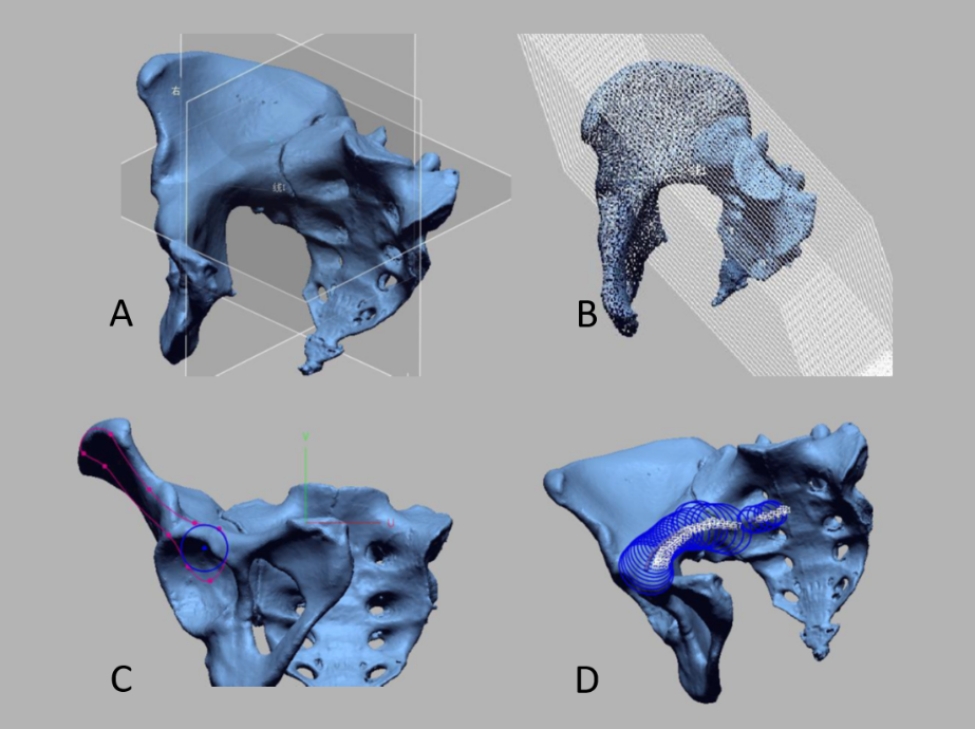
With the first sacral superior endplate plane, a three-dimensional coordinate system was established (**A**); a series of parallel cutting surfaces with a spacing of 3 mm was made in the coronal plane (**B**); the second sacral vertebra, sacroiliac joint, and iliac section outline were used as the target area (**C**); the maximum internal tangent circle was fitted to each section in the target area, and the center of the circle was marked (**D**)

With the first supra-sacral endplate plane as the horizontal plane and the pelvis symmetrical to the left and right, a three-dimensional coordinate system was established. A series of cutting planes with a spacing of 3 mm was made parallel to the coronal plane, starting from the dorsal sacral cortex and ending at the anterior superior iliac spine cortex, effectively slicing the whole pelvis into several planes. The cross-sectional profile of the second sacral vertebra, sacroiliac joint, and iliac bone was selected as the target area, and the maximum internal tangent circle was fitted to each cross-section separately, with the center of the circle marked (Fig. 1).

##### Safety domain of the S2AI screw trajectory

**Fig. 2:**
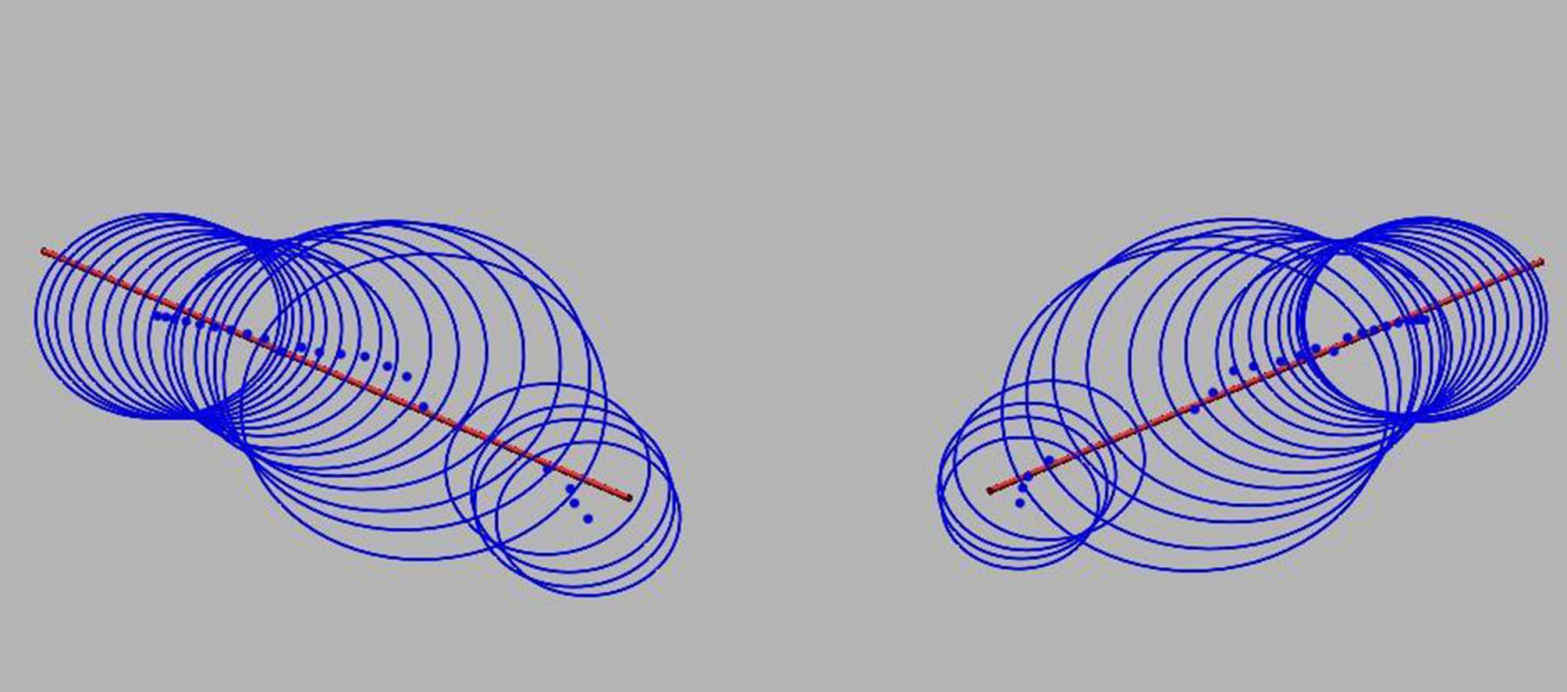
Using the straight line fitting function in Geomagic Studio software, a straight line, i.e., the axis of the safety domain, is obtained by least squares calculation for the center of the circle tangent to all sections in the safety domain

An irregular bony channel was obtained by arranging the maximum internal tangent circles of the cross-section obtained from the ipsilateral sacral vertebra, sacroiliac joint, and iliac bone in the coronal plane, respectively, which is the safety domain of the S2AI screw trajectory. The safety domain was treated as a geometry, and the line obtained by least squares calculation of the center of the scattered inner tangent circle was defined as the axis of the safety domain using the line fitting function of Geomagic Studio 12.0 software (Fig. 2).

##### Definition of the ideal screw trajectory

**Fig. 3:**
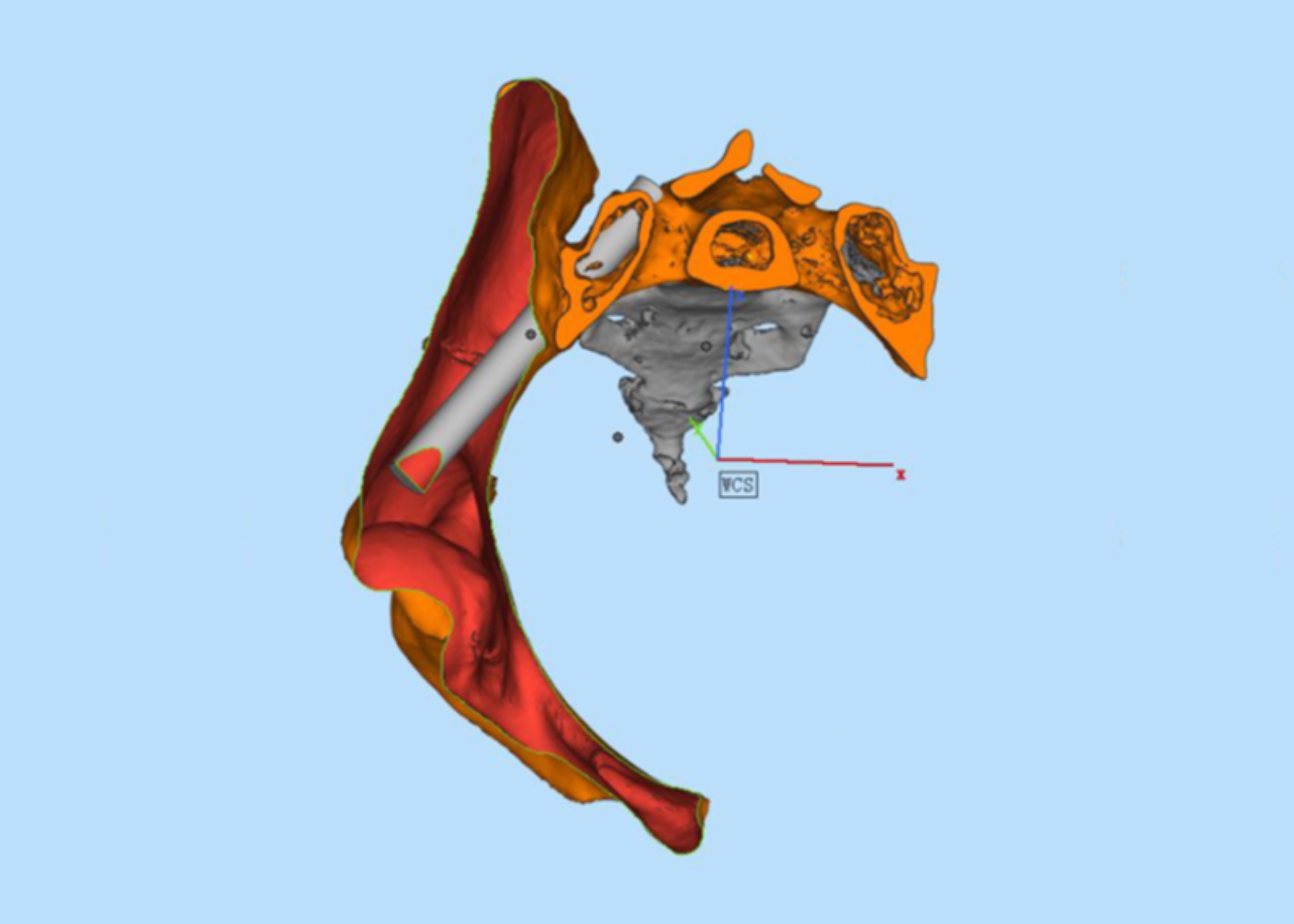
Using the Geomagic Studio 12.0 software linear fitting function, a 90 mm length axis is fitted, and the diameter is gradually increased to the first contact safety domain cortex with the axis as the center; the cylindrical model is the ideal screw trajectory

The study of the ideal screw trajectory ultimately serves for clinical screw placement. In clinical applications, the length of the S2AI screw is usually 70–90 mm, so we set the length of the screw trajectory to 90 mm (Fig. 3). In clinical applications, it is theoretically safe to place S2AI screws up to 90 mm in length.

##### The ideal screw trajectory parameters

**Fig. 4:**
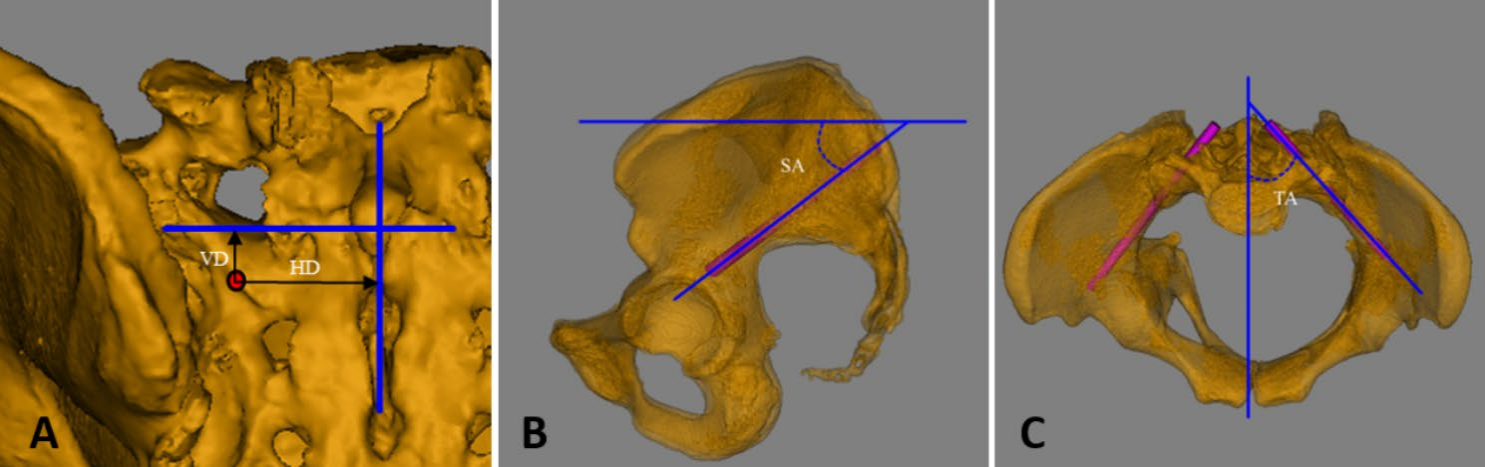
HD, the horizontal distance from the point of entry to the median sacral spine (**A**); VD, the vertical distance from the point of entry to the inferior margin of the first posterior sacral foramen; SA, the angle between the projection of the screw trajectory in the sagittal plane and the horizontal line (**B**); TA, the angle between the projection of the screw trajectory in the transverse section and the median sagittal line (**C**)

The ideal screw trajectory parameters include screw placement point, screw trajectory size, and orientation. The screw placement point is the intersection of the axis of the screw trajectory with the dorsal cortex of the second sacral vertebra. The screw placement point is located by Horizon Distance (HD) and Vertical Distance (VD); the length of the screw trajectory (L) is 90 mm, and the diameter of the screw trajectory (d) is the diameter of the cylindrical model; the direction of the screw trajectory is determined by Sagittal Angle (SA) and Transverse Angle (TA). The orientation of the screw trajectory is determined by the Sagittal Angle (SA) and Transverse Angle (TA) (Fig. 4).

#### Clinical application based on the ideal screw trajectory

We will verify the feasibility of the ideal screw trajectory in two aspects. First, the design of individualized surgical guides to assist in the placement of S2AI screws based on the ideal screw trajectory; second, the freehand screw placement based on the ideal screw trajectory parameters.

The data of 64 patients with S2AI screws placed based on the ideal screw trajectory from June 2017 to May 2021 at our hospital were selected. There were 31 male and 34 female cases, age 59.0 ± 12.7 years (range 40–82 years). They were divided into the guide group and freehand group according to the screw placement method. Postoperative CT scans were classified according to the grading method proposed by Oh et al. as Grade 0, i.e., screws did not penetrate the cortex; Grade 1, i.e., screws penetrated the cortex at a distance of less than 3 mm; Grade 2, i.e., screws penetrated between 3 and 6 mm; Grade 3, i.e., the breakthrough distance was greater than 6 mm. The excellent rate was (Grade 0/total number of screws placed), and the good rate was (Grade 0 + Grade 1/total number of screws placed).

The study was approved by the ethics committee of the First People’s Hospital of Yunnan Province, and the patients gave informed consent and signed the surgical consent form before surgery. All surgical operations were performed by the same spinal surgery team.

##### Fabrication of 3D printed surgical guide

The pelvis model was reconstructed in 3D using pelvic CT data. The ideal trajectory for the S2AI screw was designed according to the method described above. The navigation tube was determined based on this trajectory; the guide plate attachment zone was determined based on the screw placement point, and the reverse template matched with the guide plate attachment zone. The navigation tube was aligned with the reverse template to generate a virtual screw placement guide. Finally, a 3D printer was used to print out the object. Pre-operative sterilization was performed.

##### Surgical operation

The posterior median approach was used to reveal the upper fixed vertebra to the second sacral vertebra. The S2AI screws were placed with the aid of a guide plate or by hand.

For guide plate-assisted screw placement, the soft tissue in the area of the guide plate attachment was fully stripped, and the guide plate was placed firmly against the corresponding bone surface. A spherical probe was used to check the integrity of the screw trajectory, and the screw was placed after tapping.

The screw was placed freehand, and the point of entry was determined with reference to the S1 posterior sacral foramen and the median sacral spine. The direction of the screw trajectory was determined according to the sagittal and cross-sectional angles of the ideal screw trajectory, and the path was opened using an open cone. When the sacroiliac joint was reached, the joint was breached by tapping with a bone hammer. A spherical probe was used to probe the four walls and depth of the bone tract. Then, the S2AI screws were tapped and placed in the direction of the screw trajectory.

After screw placement, other operations such as decompression of the spinal canal and correction of scoliosis were performed according to the patient’s condition, and one to two drains were placed after installation of the connecting rods, and the surgical opening was closed with sutures layer by layer.

### Statistical analysis

SPSS 22.0 software was used for data analysis. The measurement data conforming to normal distribution were expressed as mean ± standard deviation. Paired χ2 samples t-test was used.

## Results

### Ideal screw placement parameters

The screw placement points [HD (4.7 ± 1.0) mm, VD (19.7 ± 1.9) mm], screw placement directions [SA (31.3°±2.3°), TA (42.4°±2.3°)], and screw trajectory dimensions (L of 90 mm, d of 13.2 ± 1.4 mm) of the ideal trajectory of S2AI screw were combined and analyzed.

### Clinical results of screw placement

128 S2AI screws with a diameter of 7.0–8.0 mm and a length of 70–90 mm were placed in 64 patients (29 in the guide group and 35 in the freehand group), and postoperative radiographs and CT scans were performed.

In the guide plate group, 58 S2AI screws were placed: 56 screws of grade 0, 2 screws of grade 1, 0 screws of grade 2, and 0 screws of grade 3. The excellent rate was 96.6%, and the reasonable rate was 100%. Postoperatively, the corresponding screw trajectory parameters [SA (33.1°±2.2°), TA (41.1°±3.4°), HD (4.4 ± 1.1) mm, VD (20.2 ± 1.8) mm] were not statistically significantly different from the ideal screw trajectory parameters, except for SA (P < 0.05) (P > 0.05, Table 1).

**Table 1:** Comparison of postoperative screw trajectory parameters with ideal screw trajectory parameters in the guide plate group

Group	SA (°)	TA (°)	HD (mm)	VD (mm)
Ideal screw trajectory	31.3 ± 2.3	42.4 ± 2.3	4.7 ± 1.0	19.7 ± 1.9
Guide template group	33.1 ± 2.2	41.1 ± 3.4	4.4 ± 1.1	20.2 ± 1.8
*t* value	-3.075	1.749	0.913	-1.141
*P* value	0.003	0.086	0.365	0.258

Note: SA, sagittal angle; TA, transverse angle; HD, horizontal distance; VD, vertical distance

In the freehand group, 70 S2AI screws were placed: 63 grade 0, 5 grade 1, 2 grade 2, and 0 grade 3. The excellent rate was 90.0%, and the reasonable rate was 97.1%. Postoperatively, the corresponding screw trajectory parameters [SA (35.9°±5.6°), TA (44.7°±3.3°), HD (5.0 ± 1.0) mm, and VD (18.4 ± 2.4) mm] were statistically different from the ideal screw trajectory parameters (P > 0.05). At the same time, HD was not statistically significantly different (P < 0.05, Table 2).

**Table 2:** Comparison of postoperative screw trajectory parameters with ideal screw trajectory parameters in the freehand group

Group	SA (°)	TA (°)	HD (mm)	VD (mm)
Ideal screw trajectory	31.3 ± 2.3	42.4 ± 2.3	4.7 ± 1.0	19.7 ± 1.9
Free-hand group	35.9 ± 3.8	44.7 ± 3.3	5.0 ± 1.0	18.4 ± 2.4
*t* value	-6.070	-3.241	-1.561	2.466
*P* value	<0.001	0.002	0.123	0.016

Note: SA, sagittal angle; TA, transverse angle; HD, horizontal distance; VD, vertical distance

There was no statistically significant difference between the two groups in terms of gender and age (P > 0.05). The excellent and good rates were higher in the guide plate group than in the freehand group, but the differences were not statistically significant (P > 0.05, Table 3). Although screws violated the cortex in both groups, there were no associated adverse events in either group.

**Table 3:** Comparison of two groups with ideal screw trajectory parameters

Group	Cases	Sex		Age (years)	Screws	Excellent rate (%)	Good rate (%)
		Male	Female				
Guide template group	29	16	14	57.8 ± 13.3	58	96.6 (56/58)	100 (58/58)
Free-hand group	35	15	20	60.0 ± 12.3	70	90.0 (63/70)	97.1 (68/70)
Statistic value	-	0.711		-0.687	-	2.083	1.683
*P* value	-	0.399		0.495	-	0.273	0.500

## Discussion

S2AI screws have similar mechanical strength to iliac screws and offer more advantages in terms of surgical trauma and postoperative complications [24–26]. Therefore, they are widely used in clinical practice and have become a hot research topic in spinal pelvic fixation. Due to the complexity of the peripelvic anatomy, screw placement deviation may damage the sciatic nerve, internal iliac artery, and other important vascular nerves, causing medically induced injuries [17, 27]. Improving the safety of screw placement remains a challenge.

Currently, various literature has reported screw placement methods for S2AI screws, among which 3D printed surgical guides, intraoperative navigation, and robotics have achieved satisfactory results in assisting screw placement [18–20]. However, robotic navigation systems are not widely available due to expensive equipment, which limits clinical applications. 3D printed surgical guide technology is relatively widespread, but preoperative planning and guide fabrication may prolong the patient’s hospital stay and increase hospital costs. Therefore, the freehand technique is still the most applied screw placement method.

Studies of the S2AI screw trajectory are mostly based on two-dimensional images, and it is worth exploring whether the three-dimensional structure can be truly reflected. In this study, the ideal trajectory for the S2AI screw was fitted using most minor squares calculations in irregular bony tracts based on three-dimensional anatomy. We arranged the maximum internal tangent circle of the cross-section obtained from the ipsilateral sacral vertebra, sacroiliac joint, and iliac bone in the coronal plane to obtain an irregular bony channel, which is called the safety domain of screw placement. Theoretically, it is always safe to insert the screw in the safety domain. The ideal trajectory for the S2AI screw is located in the “center” of the safety domain, which theoretically improves the safety of screw placement.

To verify the safety feasibility of this screw trajectory, we used a 3D printed surgical guide to assist and place the screw unassisted. We precisely placed the screw along the navigation tube of the 3D-printed surgical guide to verify the safety of the ideal trajectory. The ideal screw trajectory should have a certain amount of tolerance to provide for freehand screw placement. Therefore, we also chose freehand screw placement to further verify the feasibility of the ideal screw trajectory.

In the clinical application, a total of 128 S2AI screws were placed in 64 patients based on the ideal screw trajectory. In the guide plate group, 58 screws were placed, with an excellent rate of 96.6% (56/58) and a good rate of 100%. In the freehand group, 70 screws were placed, with an excellent rate of 90.0% (63/70) and a good rate of 97.1% (68/70). The results indicate that either guide plate assisted screw placement or freehand screw placement is safe and feasible based on rational access. Both the excellent and good rates were higher in the guide plate group than in the freehand group, although the difference between them was not statistically significant (P > 0.05), but still clinically significant. When comparing the postoperative screw trajectory parameters with the ideal screw trajectory parameters, there was no statistically significant difference between the guide plate group except for SA. In contrast, the differences between parameters in the freehand group were statistically significant, except for HD. This demonstrates the greater precision of guide plate assisted screw placement. The good rate of 97.1% for freehand screw placement, despite some differences with the ideal screw trajectory parameters, indicates that freehand screw placement based on the ideal screw trajectory has a better tolerance for errors and reflects the safety feasibility of the ideal screw trajectory.

In this study, the ideal trajectory of S2AI screw was designed and achieved satisfactory results, but there are still limitations. Limitations of the screw trajectory design include: (1) The number of cases selected for the design of the S2AI screw trajectory was relatively small, which may have biased the results to some extent. (2) We set the screw trajectory length to 90 mm, which may be hidden dangers when inserting screws exceeding 90 mm, although this is rare in clinical practice. O’Brien [28] found no biomechanical difference between 65 mm S2AI screws and 80 mm S2AI screws in a biomechanical study. Therefore, we believe that setting the screw trajectory length at 90 mm is appropriate. (3) This study is based on a normal structure adult pelvis study, which is not applicable in the clinical setting for patients with pelvic deformities. Screw placement with the assistance of 3D printed surgical guides, intraoperative navigation, or robotics is recommended in this case. The limitations of screw trajectory validation are mainly in the relatively insufficient sample size and single-center studies, which need to be increased for future clinical applications and multicenter studies.

In conclusion, the present study is based on a three-dimensional anatomical structure, and the ideal screw trajectory for the S2AI screw was fitted using least squares calculations in an irregular bony channel with safe feasibility in clinical applications. It provides a reference basis for freehand screw placement.

## Conclusion

The S2AI screw-based ideal trajectory placement is a safe, feasible and accurate method of screw placement.

## Data Availability

The data used to support the findings of this study are available from the corresponding author upon reasonable request.

## Abbreviations

S2AI: S2-alar-iliac
CT: Computed tomography
DICOM: Digital imaging and communications in medicine
STL: Stereolithograph
SA: Sagittal angle
TA: Transverse angle
HD: Horizontal distance
VD: Vertical distance
